# *Dermatophagoides pteronyssinus* immunotherapy changes the T-regulatory cell activity

**DOI:** 10.1038/s41598-017-12261-2

**Published:** 2017-09-20

**Authors:** M. Gonzalez, I. Doña, F. Palomares, P. Campo, M. J. Rodriguez, C. Rondon, F. Gomez, T. D. Fernandez, J. R. Perkins, M. M. Escribese, M. J. Torres, C. Mayorga

**Affiliations:** 1 Research Laboratory-Allergy Unit, IBIMA-Regional University Hospital of Malaga, UMA, Malaga, Spain; 2 Allergy Service, IBIMA-Regional University Hospital of Malaga, UMA, Malaga, Spain; 3 Institute for Applied Molecular Medicine (IMMA), School of Medicine, Universidad CEU San Pablo, Madrid, Spain; 4 Basical Medical Sciences Department, Faculty of Medicine, CEU San Pablo University, Madrid, Spain

## Abstract

Subcutaneous specific immunotherapy (SCIT) has been shown to modify the *Dermatophagoides pteronissinus* (DP) allergic response, characterized by generation of Treg cells. However, studies have reported no changes in the proportion of Treg cells after immunotherapy, indicating that the effects may be due to modifications in their regulatory activities. We aimed to determine whether Tregs generated by DP-SCIT can switch the allergic response to tolerant and study the involvement of suppressive cytokines on it. Twenty-four DP-allergic rhinitis patients were recruited, 16 treated with DP-SCIT and 8 untreated. Treg and T effector cells were isolated before and after DP-SCIT, and cocultured in different combinations with α-IL-10, α-TGF-β blocking antibodies and nDer p 1. Treg cells after DP-SCIT increased Th1 and decreased Th2 and Th9 proliferation. Similarly, they increased IL-10 and decreased IL-4 and IL-9-producing cells. α-IL-10 affected the activity of Treg cells obtained after DP-SCIT only. Finally, DP-specific IgG4 levels, Treg percentage and IL-10 production were correlated after DP-SCIT. These results demonstrate that DP-SCIT induces Treg cells with different suppressive activities. These changes could be mediated by IL-10 production and appear to play an important role in the induction of the tolerance response leading to a clinical improvement of symptoms.

## Introduction

Allergic rhinitis (AR) can affect up to 30% of the population and its prevalence is increasing^1^. Frequently, AR presents comorbidities such as allergic conjunctivitis and other respiratory disorders like rhinosinusitis and asthma^2,3^. It is typically caused by common aeroallergens derived from grass, birch, pet or house dust mites^4,5^. It can greatly affect quality of life and be a burden to public health systems^6^ due in part to the cost of pharmacological control of symptoms.

Adaptive immune reactions can occur in AR, being T lymphocytes, particularly Th2 (producing IL-4, IL-5 and IL-13, amongst others) and Th9 cells (producing IL-9 and IL-10)^7^ the main effector T cells (Teff). These cells have an essential role in the induction of other effector cells involved in AR-related inflammation, such as mast cells, basophils and eosinophils^8,9^.

Specific immunotherapy (sIT) with allergens such as grass pollen or *Dermatophagoides pteronyssinus* (DP) is an effective treatment for IgE-mediated allergic respiratory diseases^10^, inducing long-term clinical benefits and immunological tolerance^11,12^. The underlying immunological mechanisms of sIT include immune deviation from a Th2 to Th1 cell pattern^13^, blocking antibody production^14^ and regulatory T cell (Treg) induction^15,16^. Moreover, as shown for DP subcutaneous immunotherapy (DP-SCIT) in patients with AR, clinically effective sIT is correlated with immunological changes at both the humoral and cellular levels. The former consists of a decreased ratio of specific IgE/IgG4 and an increase in sIgG4. The latter consists of a decreased effector cell response for cells such as Th2, Th9 and Th17 as well as inflammatory plasma cells, and increased levels of Th1 cells^17,18^. Similar results have been found at the humoral level for DP-SCIT in patients with the AR subphenotype, local AR^19^.

It is well known that allergen-specific Tregs have an important role in the immunological induction of tolerance observed during sIT. They are able to inhibit the activation, proliferation and effector functions of a wide range of target cells including innate cells, antigen-presenting cells and Teff cells (mainly Th2 and Th9)^20^. Tregs also release cytokines such as IL-10 and TGF-β, which have key suppressive activities^21,22^. Different subsets of Tregs have been described depending on their origin: the natural Tregs (nTreg), which originate in the thymus and are the major cell population for the control of immune self-tolerance; and induced Tregs (iTreg), which are peripherally generated from naïve CD4^+^ T-cells in response to foreign antigens, during sIT and also *in vitro* ^23^. These Treg subsets use different suppression mechanisms: while nTregs predominantly use cell-cell contact-dependent mechanisms, iTreg suppression is generally performed via immunomodulatory cytokines such as IL-10 and TGF-β^24^. Tregs are characterized by the Foxp3 protein, which is a member of the forkhead transcription factor family. This protein plays an important role in the regulation of the expression of genes involved in many Treg cell processes^25^.

Although several studies have attributed the beneficial clinical effects during sIT to an increase in the Treg population^26,27^, other studies did not find this change^28,29^. Moreover, many authors claim that the efficacy is largely related to changes in the number of IL-10 producing T-cells^30^, suppressing Th2 production of IL-4 and leading to reduced IgE production by plasma cells^17^. We postulate that various mechanisms of regulation occur during sIT, affecting the function of allergen-specific-Tregs and leading to the suppression of the Th2-type response. Therefore, the effectiveness of sIT could be due not only to the increased percentage of Treg cells but also to their enhanced activity. Whether a reduced capacity of Tregs to suppress the allergen-specific T-cell response in allergic-patients is modified during sIT needs to be established. We hypothesise that DP-SCIT will induce Tregs  with a high suppressive activity that acts on effector Th2 cells, shunting the immunological profile towards a Th1/Treg pattern and a tolerant response. To explore this, we have determined the changes in Treg activity produced after 1-year of DP-SCIT in a group of patients with AR to DP compared to a group of untreated patients. More specifically, we examined the proliferation of Th1/Th2/Th9 cells and IL-10, IL-4 and IL-9 cytokine producing-cells in different combinations of Treg and Teff cells, before and after DP-SCIT, as a primary endpoint. We also examined the effects of IL-10 and TGF-β on the proliferation of Th1/Th2/Th9 cells as a secondary endpoint.

## Results

Twenty-four patients with AR were included, 16 were to receive DP-SCIT and 8 to remain untreated as controls. The median age of all subjects was 28 (22–30). A total of 8 subjects (33.33%) were female. No significant differences were found in terms of clinical characteristics (sex, age, asthma symptoms, rinoconjunctivitis clinical score symptoms (RCSS), skin prick test (SPT), intradermal test (IDT) and nasal provocation test (NPT)) between groups at baseline. No patients presented any systemic adverse symptoms during DP-SCIT.

After 1 year of DP-SCIT, we observed a significant decrease in IDT area (p = 0.041) and a reduction of RCSS score (p = 0.036). On the contrary, during this period of time, untreated patients showed an increased IDT area and a significant decrease (p = 0.037) in the DP concentration needed to induce a positive NPT, indicating higher reactivity (Table 1).

**Table 1:** Changes in clinical characteristics of patients after one year of DP-SCIT.

	0 months	12 months	p
**Treated patients (n = 16)**			
RCSS	2.1 (0.46–11.85)	1.55 (0.82–4.57)	0.036
Medication score	0.52 (0–4.67)	0.25 (0–2.5)	0.16
IDT DP (mm^2^)	168 (64–225)	100 (48–190)	0.041
Concentration DP (mg/ml) at positive NPT	0.04 (0.04–4)	0.04 (0.04–0.4)	0.713
**Untreated patients (n = 8)**			
RCSS	4.745 (0.5–6.14)	5.53 (0.92 –7.6)	0.5
Medication score	0.07 (0–0.5)	0.46 (0–2.03)	0.458
IDT DP (mm^2^)	132 (110–324)	143 (70–225)	0.716
Concentration DP (mg/ml) at positive NPT	0.4 (0.04–4)	0.04 (0.04–0.04)	0.037

RCSS: rhinoconjunctivitis symptom score; IDT: Intradermal test; DP: *Dermatophagoides pteronissinus*; NPT: Nasal provocation test. Data are represented as medians and range. Statistical analysis for two related samples were carried out by Wilcoxon test; values p ≤ 0.05 are considered statistically significant.

### DP-SCIT induces changes in the DP-specific immunoglobulin pattern and stimulates Treg generation

**Figure 1:**
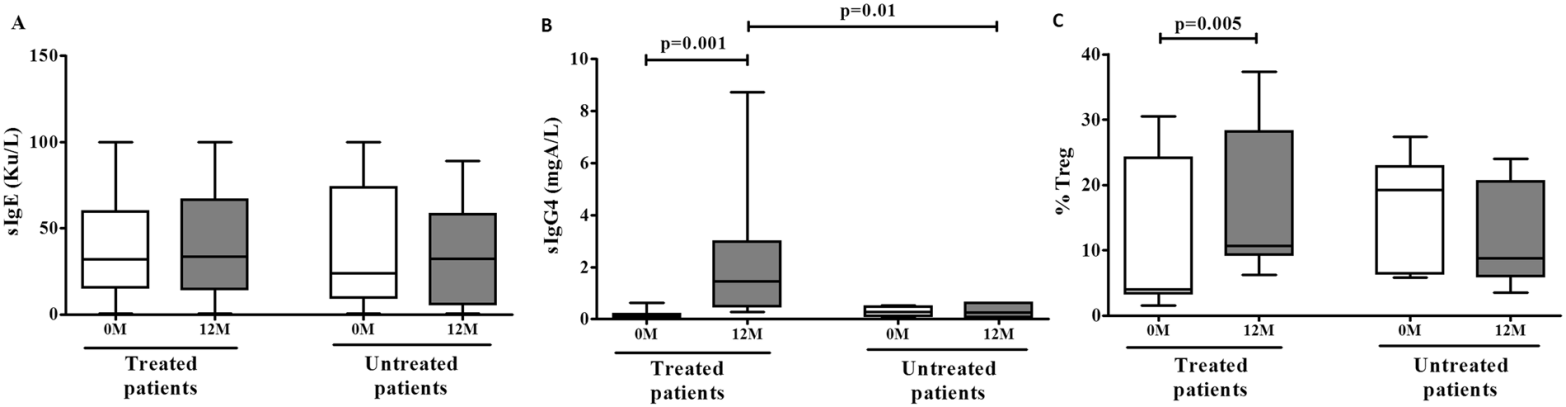
Determination of changes in DP-specific immunoglobulins and Treg populations. (**A**) DP-sIgE (Ku/L) and (**B**) DP-sIgG4 (mgA/L) levels for treated and untreated patients before (0 M) and after (12 M) 1-year of DP-SCIT. (**C**) Comparative analysis of treated (N = 16) and untreated (N = 8) patients before (0 M) and after (12 M) 1-year of treatment. Statistical Wilcoxon (related samples) and Mann-Whitney tests (non-related samples) were performed; p-values ≤ 0.05 are considered statistically significant.

The effects of DP-SCIT on DP-specific immunoglobulin production were evaluated. For this, DP-sIgE and sIgG4 levels were measured in sera samples. No changes were observed for DP-specific IgE (Fig. 1A), however a significant increase in DP-specific IgG4 levels was seen at 12 months (p = 0.001) for treated patients only. Moreover, when we compared treated and untreated patients, although no differences in DP-sIgG4 levels were found between groups for samples taken at 0 months, significantly higher levels of DP-sIgG4 were observed at 12 months (p = 0.01) (Fig. 1B).

**Figure 2:**
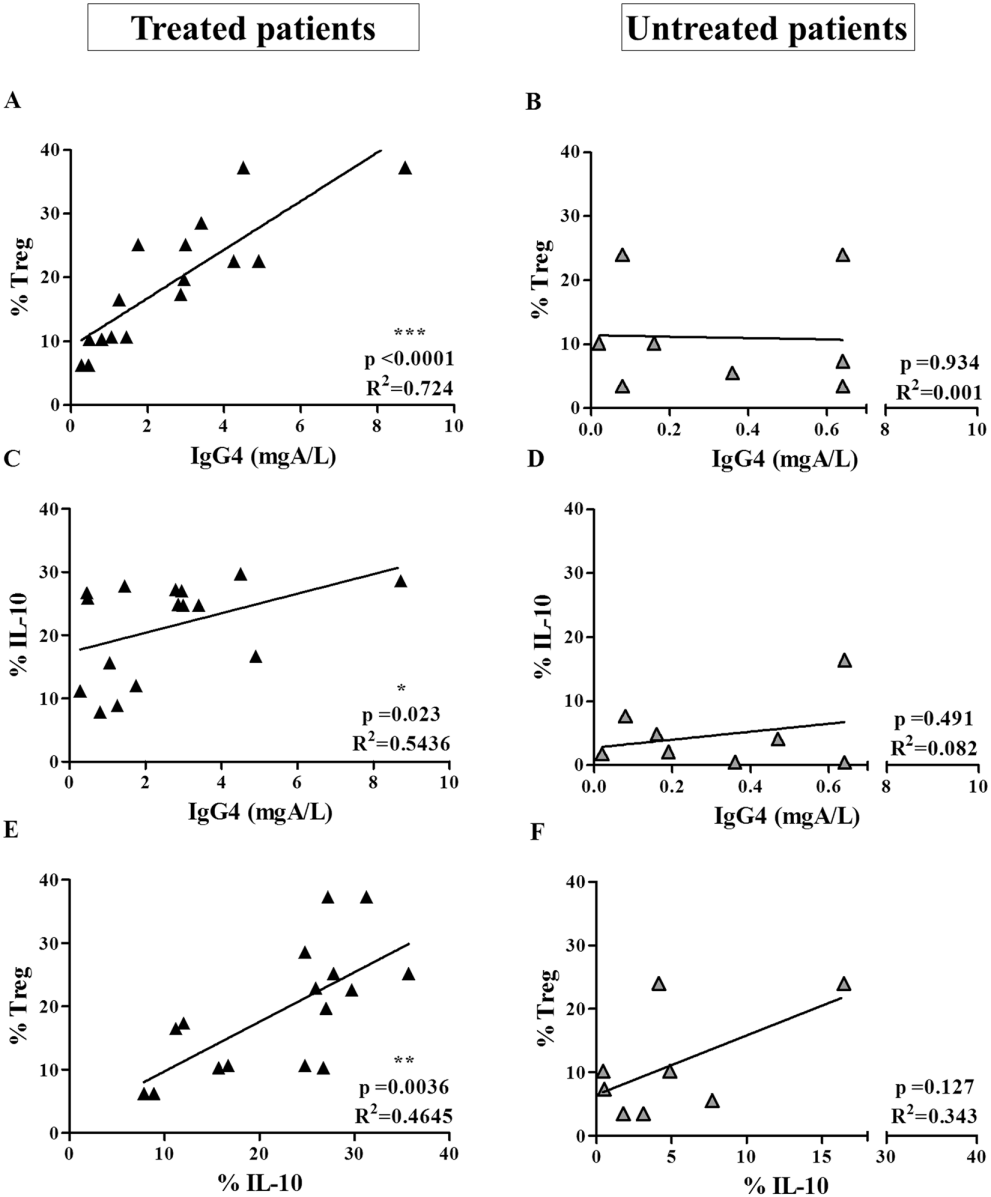
DP-sIgG4 production after 12 months of DP-SCIT correlates with IL-10 production and iTregs. Correlations between percentage of Treg and DP-sIgG4 levels (mgA/L) at 12 months in treated (N = 16) (**A**) and untreated (N = 8) patients (**B**). Correlations between percentage of IL-10 secreting cells and DP-sIgG4 levels (mgA/L) at 12 months in treated (N = 16) (**C**) and untreated (N = 8) patients (**D**). Correlations between percentage of Treg and percentage of IL-10 secreting cells at 12 months in treated (N = 16) (**E**) and untreated (N = 8) patients (**F**). Statistical analysis was carried out using the Pearson correlation coefficient; p-values ≤ 0.05 are considered statistically significant.

We detected a significant increase in the percentage of Treg cells in peripheral blood mononuclear cells (PBMCs) at 12 months for treated patients (p = 0.005) (Fig. 1C). This can be considered a potential specific marker of successful sIT^18^. We also found a significant positive correlation of DP-sIgG4 levels with the percentage of Treg cells (Fig. 2A) and IL-10 producing cells (Fig. 2C), and between the percentage of Treg cells and IL-10 producing cells (Fig. 2E) for treated patients after 12 months of DP-SCIT (R^2^ = 0.724, p < 0.0001; R^2^ = 0.5436, p = 0.023 and R^2^ = 0.464, p = 0.0036 respectively). No correlations were found for untreated patients (Fig. 2B,D and F).

### The proliferative response to DP by Teff cells is modified by Treg cells induced during DP-SCIT

**Figure 3:**
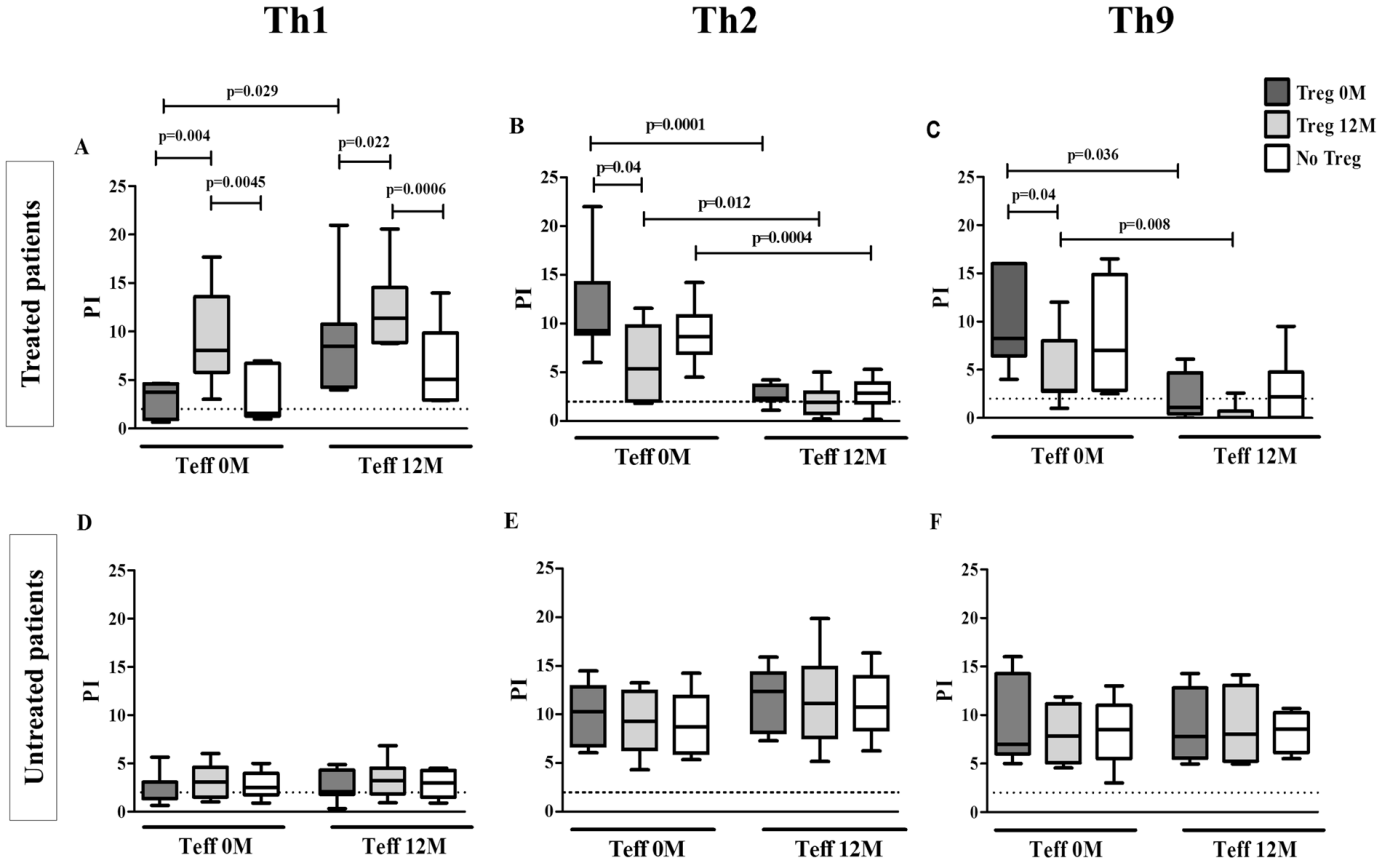
Proliferative response of Teff after DP stimulation in absence or presence of Treg cells. Box plots represent comparative analysis of proliferation index (PI) of different T cell subsets Th1 (**A**), Th2 (**B**) and Th9 (**C**) from treated (N = 16) and Th1 (**D**), Th2 (**E**) and Th9 (**F**) from untreated (N = 8) patients in different combinations of Teff and Treg at different times: 0 or 12 months (0 M and 12 M, respectively). Statistical analysis was carried out by Wilcoxon test; p-values ≤ 0.05 are considered statistically significant. PI: proliferative index.

In order to analyze changes in Treg functionality during DP-SCIT, we determined Treg-mediated changes in the proliferative response to DP for different T cell subpopulations. For this purpose, we co-cultured Tregs obtained at 0 and 12 months, with effector T-cells from both time points and determined the proliferative response of these Teff subpopulations. In the treated group, we found that Treg cells obtained at 12 months led to increased Th1 proliferation compared with Treg cells obtained at 0 months and in the absence of Tregs, in cocultures with Teff cells obtained at both 0 months (p = 0.004 and p = 0.0045, respectively) and 12 months (p = 0.022 and p = 0.0006, respectively) (Fig. 3A). Moreover, Treg cells after 12 months led to decreased Th2 and Th9 proliferation compared with Treg cells from 0 months, in cocultures with Teff cells obtained only at 0 months (p = 0.04 in both cases) (Fig. 3B,D, respectively for Th2 and Th9).

Regarding the effects of the immunotherapy on Teff cells, we observed a significant increase of Th1 proliferation at 12 months in the treated group when cocultured with Treg cells from 0 months (p = 0.029) (Fig. 3A). The opposite trend was found for Th2 and Th9 cells (p = 0.0001 and p = 0.036, respectively). In addiction Th2 and Th9 proliferation decreased between 0 and 12 months when cocultured with 12 months Treg cells (p = 0.012 and p = 0.008, respectively) (Fig. 3B,C). Th2 proliferation also decreased between 0 and 12 months in the absence of Tregs (p = 0.0004) (Fig. 3B). No changes were observed for untreated patients (Fig. 3D,E and F). Similar results had been published by our group^18^.

### The pattern of effector T cell cytokine production in response to DP is modified by Tregs induced by DP-SCIT

**Figure 4:**
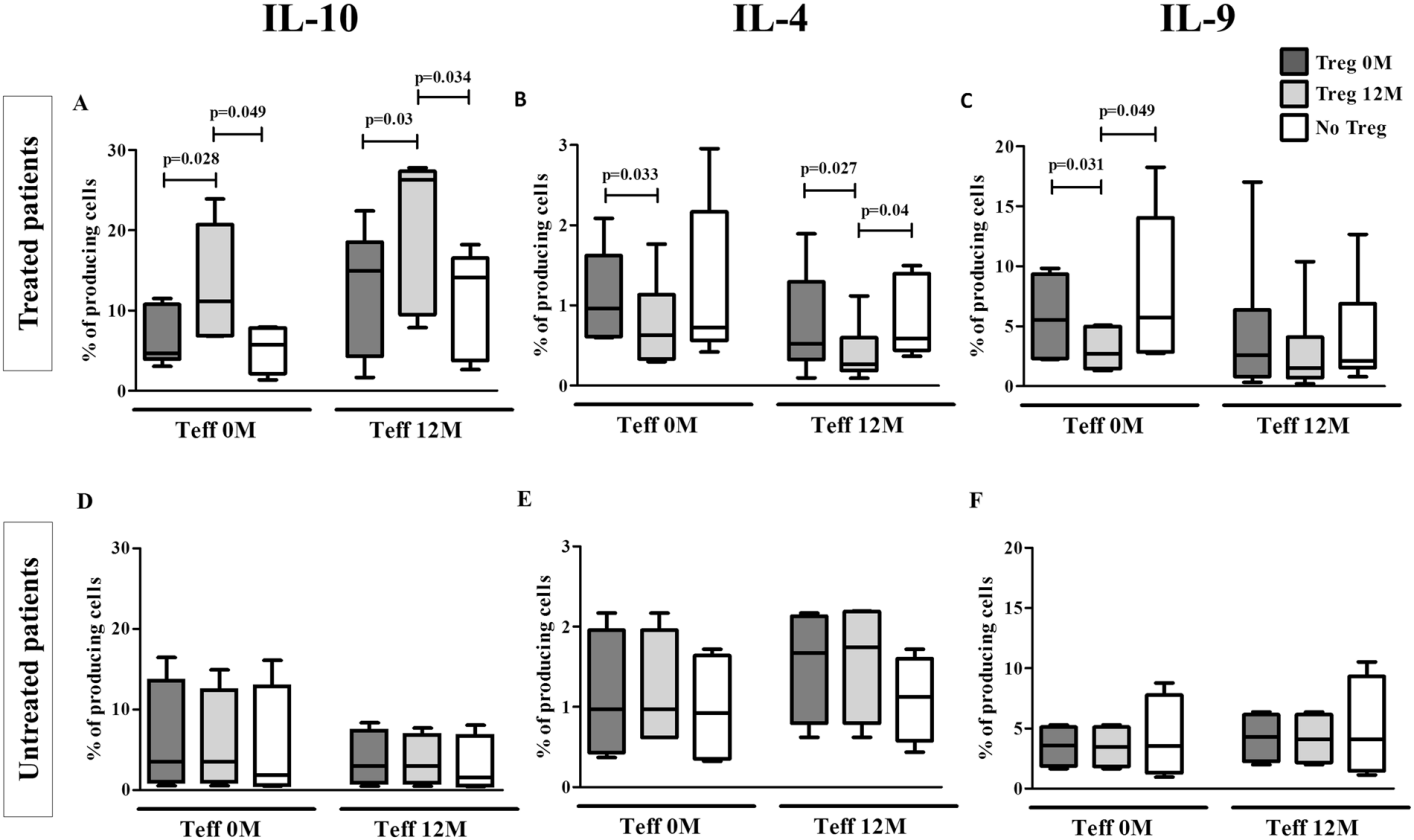
Cytokines  produced by Teff cells after DP stimulation in absence or presence of Treg cells. Box plots represent comparative analysis of percentages of Teff cells producing IL-10 (CD3^+^CD4^+^IL-10^+^) (**A**), IL-4 (CD3^+^CD4^+^IL-4^+^) (**B**) and IL-9 (CD3^+^CD4^+^IL-9^+^) (**C**) from treated (N = 16) and IL-10 (CD3^+^CD4^+^IL-10^+^) (**D**), IL-4 (CD3^+^CD4^+^IL-4^+^) (**E**) and IL-9 (CD3^+^CD4^+^IL-9^+^) (**F**) from untreated (N = 8) patients in different combinations of Teff and Treg at 0 or 12 months (0 M and 12 M, respectively). Statistical analysis was carried out by Wilcoxon test; p-values ≤ 0.05 are considered statistically significant.

We analyzed the effect of Tregs on cytokine production before and after treatment. For the treated group, we found that Treg cells obtained at 12 months increased the percentage of IL-10-producing cells compared with Tregs obtained at 0 months and in absence of Treg, for cocultures with Teff cells obtained both at 0 months (p = 0.028 and p = 0.049, respectively) and 12 months (p = 0.03 and p = 0.034, respectively) (Fig. 4A).

Moreover, Treg cells at 12 months led to a significant decrease in the percentage of IL-4-producing cells in coculture with Teff cells at both 0 and 12 months, compared to the effect induced by Tregs at 0 months (p = 0.033 and p = 0.027, respectively). Similar results were found comparing Teff cells at 12 months in the absence of Treg cells (p = 0.04 for Teff at 12 months) (Fig. 4B). Similarly, Treg cells at 12 months led to a lower percentage of IL-9-producing cells compared to Treg of 0 months and in the absence of Treg, in cocultures with Teff cells obtained at 0 months (p = 0.031 and p = 0.049, respectively) (Fig. 4C). No changes in the percentage of cytokine-producing cells were observed for untreated patients (Fig. 4D–F).

### The iTreg suppressive activity is IL-10-dependent

Using blocking antibodies (α-IL-10 and α-TGF-β), we tried to elucidate the potential role of these cytokines in the suppression of the allergic response induced during 1 year of DP-SCIT, and their effects on the proliferation of T-cell subsets.

**Figure 5:**
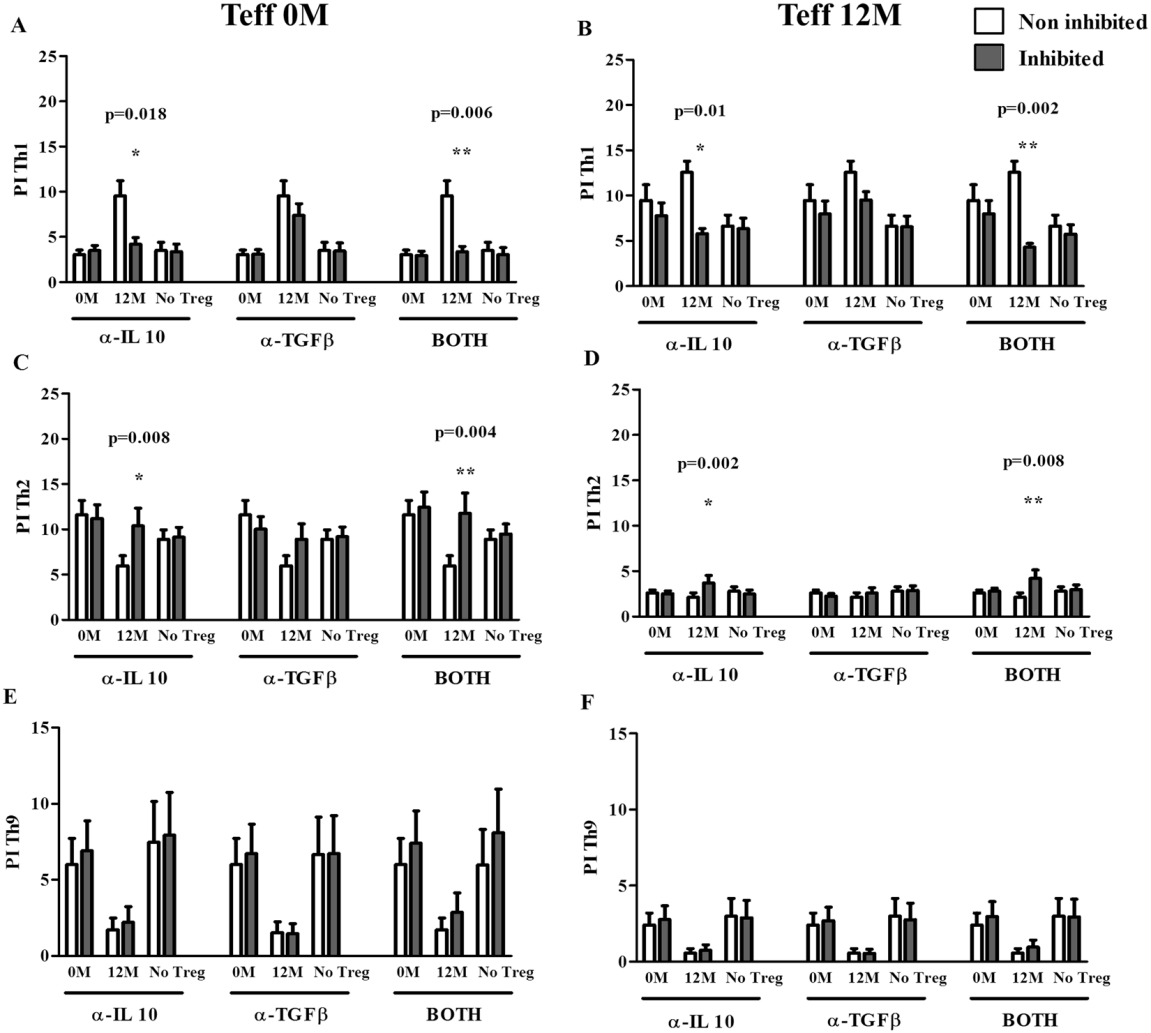
Changes induced by α-IL-10 and α-TGF-β blocking antibodies in different subpopulations of DP-specific Teff cells from DP-SCIT treated patients (N = 16). Bars represent different comparisons of IL-10 and TGF-β inhibitions performed in absence or presence of Tregs (from 0 or 12 months) on Th1 0 (**A**) and 12 months(**B**), Th2 0 (**C**) and 12 months (**D**) and Th9 0 (**E**) and 12 months (**F**). Statistical analysis was carried out by Wilcoxon test; p-values ≤ 0.05 are considered statistically significant. PI: proliferative index; 0 M: Treg cells obtained at 0 months; 12 M: Treg cells obtained at 12 months.

Results indicated that the presence of these inhibitors only affected the Teff cultures taken at 0 and 12 months, from treated patients and when cocultured with Tregs at 12 months. Data showed that Th1 proliferation was significantly reduced for both 0 and 12 months Teff cells with the presence of α-IL-10 both alone (p = 0.018, p = 0.01 respectively) and together with α-TGF-β (p = 0.006, p = 0.002 respectively) (Fig. 5A,B). The opposite pattern was seen for Th2 proliferation: Th2 levels increased for both 0 and 12 month Teff cells in the presence of α-IL-10, both alone (p = 0.008 for Teff at 0 months and p = 0.002 for Teff at 12 months) and together with α-TGF-β (p = 0.004 for Teff at 0 months and p = 0.008 for Teff at 12 months) (Fig. 5C,D). Moreover, although a tendency to an increased Th9 proliferative response could be observed when Teff 0 M and Treg 12 M were cocultured in the presence of α-IL-10, no significant differences were found. No changes were observed for untreated patients (Fig. S1).

## Discussion

Historically, the clinical effect of allergen-sIT has been linked to immunological changes such as increased levels of sIgG4 blocking antibodies^31^, accompanied by the suppression of allergen-specific Th2 cell responses by Tregs^30,32^.

Although several studies into the effects of Tregs on sIT have been carried out^24,33^ and the generation of CD4^+^CD25^+^IL-10^+^ Treg cells after sIT has been reported^16^, the changes in the suppressive activity of these cells brought about during treatment with a specific allergen had not been analyzed until now. The goal of this study was to assess whether sIT can affect Treg suppressive activity in order to redirect the allergic response to a tolerant one. This was evaluated by analyzing Tregs before and after 12 months of DP-SCIT and the immunological changes that these cells induced in terms of a DP-specific proliferative cell response and their cytokine expression patterns. One year of treatment led to a decrease in IDT area. This agrees with previous studies using grass-SCIT^34^ and DP-SCIT^18,19^.

Additionally, we found changes in Treg quantity and function, at both the humoral level, evidenced by changes in antibody levels, and the cellular level, evidenced by changes in Th cell subsets and cytokine production.

These responses are closely related, in fact Tregs can interact with B-cells by an IL-10 mediated mechanism, stimulating IgG4 production and inhibiting IgE production^35^. In previous studies^17^, we found that increases in the number of IgG4 secreting cells and sIgG4 levels were linked to an improvement of the clinical response to DP-SCIT. Moreover, although no changes in sIgE levels were found here, a significant increase in serum sIgG4 and in the percentage of Treg cells was observed in treated patients, in agreement with other studies^36,37^. Our findings also revealed a significant correlation between Treg-cell percentage, DP-sIgG4 levels and IL-10 production after DP-SCIT for treated patients, as has been shown previously^38,39^. These data suggest that during DP-SCIT there is an increase in iTreg cells that produce IL-10, which is involved in modulating the immunoglobulin secretion pattern towards sIgG4, blocking sIgE^40^.

It is generally accepted that Tregs play a key role in the induction of tolerance during sIT. Previous studies found an increase in the percentage of these cells during treatment^18,26,27,41^, however, others did not^28,29^. Moreover, it has been observed that Treg cells from non-sensitized donors have a higher capacity to suppress allergen-specific proliferative responses compared to Treg from sensitized individuals^42^. These data suggest the involvement of other regulatory mechanisms related to the function of Treg cells during the sIT. In keeping with this, epigenetic changes in Tregs have been shown to influence their suppressive functions and thus could play a role in the generation of immune tolerance^32,43^. We found that iTregs from treated patients led to changes in the DP-specific proliferative response. Moreover, these iTregs from treated patients were able to increase the percentage of IL-10 secreting T cells, probably due to the IL-10 production by iTreg cells. Similar results have been found for sublingual DP-sIT^44,45^. Taken together, these results suggest that sIT can induce iTreg, with higher suppressive activity compared to nTreg, and indicates a key role for these cells in switching an allergic response to tolerance^46,47^.

The iTregs present several differences compared to nTregs^48,49^. They carry out their suppressive activity through the secretion of cytokines such as IL-10 and TGF- β^27,50^. IL-10 can inhibit proinflammatory cytokines and IgE production and inducing IgG4 secretion^51^. On the other hand, TGF-β has been shown to inhibit proinflamatory cytokines production, promote IL-10 production by T-cells^52^ and induce Treg cells^53,54^. In order to elucidate whether the suppressive activity of iTregs is mediated by IL-10 or TGF- β, we analyzed changes in the T cell proliferative response in the presence or absence of cytokine-neutralizing antibodies. We found that the previously mentioned effects were only inhibited significantly when α-IL-10 was present. This neutralizing antibody inhibited Th1 proliferation and blocked the suppression of the proliferative responses of Th2 in treated patients. These results consistently match data from other authors for sublingual sIT-treated patients^21,55^.

Moreover, when both cytokines were blocked simultaneously, no differential effects were observed compared to blocking IL-10 alone, suggesting there is no cooperative effect between the two cytokines.

There are some studies indicating the role of TGF-β in Treg activity^56^, however in our study the TGF-β mediated mechanism does not seem to have an important effect. These discrepancies may due to the time of performance of each study, since the effect of TGF-β-mediated T-cell suppression may be more important during a relatively early phase of the treatment (for example, after 6 months of sublingual sIT) and not at later time points (12 and 24 months)^56^. Our results are in line with those published by Bohle and colleagues^57^, who showed that IL-10 but not TGF-β mediated suppression of allergen-specific T-cell proliferation occurs after birch pollen sublingual sIT.

One limitation of this study is due to the small number of available cells, which limited the number of cytokines we were able to measure. Therefore, we chose cytokines that were representative of each cellular subset (IL-10→Tregs, IL-4→Th2 and IL-9→Th9) in order to achieve as broad a vision of the changes in the immune response as possible. For the same reason, have been unable to study the cell-cell interaction effects using transwell plates to isolate the cells from each other. In any case, we believe the most important factor underlying the suppressive mechanisms of iTregs is cytokine mediated interaction.

In conclusion, our study revealed that DP-SCIT induces Treg cells with a high capacity to suppress the allergic response. The switch in immunologic response towards tolerance during DP-SCIT has been shown to involve the down-regulation of Th2 responses produced by iTreg cells via an IL-10-mediated mechanism. This suppression leads to decreased levels of Th2 cytokines (i.e. IL4 and, IL13) that are involved in the inhibition of Th1 generation^58–61^. Although more investigation is needed, the iTregs with IL-10-mediated suppressive action represent promising targets for future allergy therapies, and potential efficacy biomarker for novel types of allergen-specific immunotherapy and cell-based therapies.

## Methods

### Patient work-up

We included patients with a history of persistent AR to DP. Patients were diagnosed by SPT, NPT and having DP-specific IgE levels higher than 0.35 Ku/L (ImmunoCAP-FEIA). Patients were treated with SCIT to DP (Acaroid^©^, Allergopharma KG, Reinbeck, Germany) for 12 months using a conventional schedule^17^.

To assess the clinical improvement in response to DP-SCIT, both RCSS and medication score were calculated as described^18^. A group of patients with AR to DP but not treated with DP-SCIT and with similar clinical characteristics to the treated patients was included as a control.

The study was conducted in accordance with the declaration of Helsinki, all patients participating in the study gave their informed consent and all protocols were approved by the institutional ethical committee (*Comité de Ética de la Investigación Biomédica Provincial de Málaga*).

### Skin tests

SPT were performed with a battery of 17 common inhalant allergens, including pollens, DP, molds and animal danders (ALK, Madrid, Spain). IDT was carried out with freshly reconstituted freeze dried DP (0.4 mg/ml) (ALK, Madrid, Spain) as previously described^62^ and results expressed as mm^2^. IDT to DP was performed at 0 and 12 months in order to assess clinical improvement.

### Nasal provocation test

NPT was carried out at 0 and 12 months in both treated and untreated groups as described previously^63^. Briefly, symptom-free patients [total visual analogue scale (VAS), <60 mm] were intranasally challenged with two puffs (100 mL) of saline in each nostril to exclude nasal hyperreactivity. If this result was negative, 15 min later we began administering reconstituted freeze-dried allergen solutions of DP (0.0, 0.4 and 4 mg/mL) at 15-min intervals. Two puffs (100 mL) of the solution at room temperature were applied in each nostril. Responses were monitored by means of acoustic rhinometry (SRE 2000 rhinometer; Rhinometrics, Lynge, Denmark) and symptom score. Acoustic rhinometry was performed following the guidelines of the Standardization Committee on Acoustic Rhinometry (E4), measuring the volume of the nasal cavity that corresponds to the lower turbinate (VOL 2–6 cm) in each nostril. Nasal-ocular symptoms, including obstruction, rhinorrhea, itching and sneezing were monitored at each time-point by placing a vertical mark on a horizontal VAS of 100 mm. The total range of the VAS during NPT was 0–500 mm. The response to nasal challenge was evaluated based on subjective (VAS of nasal-ocular symptoms) and objective (VOL 2–6 cm) parameters. A NPT was considered positive when an increase greater than 30% in the VAS score and a decrease greater than 30% in nasal cavity volume was observed, compared to the baseline value.

### Sample collection and storing

Peripheral blood samples were collected from treated and untreated patients, at 0 and 12 months and processed immediately. PBMCs were isolated by Ficoll-Paque density gradient (Pharmacia Biotech, Barcelona, Spain) and frozen in RPMI 1640 medium supplemented with 2 mM of L-glutamine (BioWhittaker, Pittsburgh, PA, USA), gentamicin (5 mg/ml) (Normon, Madrid, Spain), streptomycin (50 ng/ml), penicillin (100 IU/ml), 40% of fetal bovine serum (BioWhittaker) and 10% of DMSO (Sigma, St. Louis, MO, USA) and stored in liquid nitrogen.

Serum was collected for specific IgE (sIgE) and IgG4 (sIgG4) determination, and stored at −20 °C.

Samples were managed by the Málaga Hospital-IBIMA Biobank, which belongs to the Andalusian Public Health System Biobank.

### Specific IgE and IgG4 determination

Levels of sIgE and sIgG4 to DP (d1) were determined by ImmunoCAP-FEIA in serum samples, according to the manufacturer’s instructions (Thermo Fisher Scientific, Massachusetts, USA).

### Treg cells determination

The percentage of Treg cells (CD3^+^CD4^+^CD25^+^CD127_low_) (Fig. S2) during DP-SCIT was determined in PBMCs at 0 and 12 month using specific MoAbs: CD3-PerCP, CD4-APC (BD™ Biosciences), CD25-PECy7 and CD127-PE (Biolegend^®^, San Diego, CA, USA). Cells were phenotyped using a BD^TM^ FACS Canto II flow cytometer (BD^TM^ Biosciences). Results were analyzed with FlowJo^®^ software (Tree Star, Inc, Ashland, OR, USA) and dead cells were excluded using Near-IR fluorescent LIVE/DEAD^®^ Fixable Dead Cell dyes (Thermo Fisher Scientific).

### Cell isolation

Treg and Teff cells were isolated from PBMCs at 0 and 12 month using the immunomagnetic EasySep^TM^ Human CD4^+^CD127^low^CD25^+^ Regulatory T Cell Isolation Kit (STEMCELL^TM^ Technologies, Vancouver, Canada).

### Analysis of the suppressive capacity of Treg cells during DP-SCIT

Teff cells were obtained at 0 and 12 months and cocultured at a 5:1 ratio with Tregs from 0 and 12 months, respectively. The 5:1 ratio was chosen based on previous studies^56^. Teff cells were also cultured alone (without Treg) as a control. These cultures were then stimulated with nDer p 1 (10 µg/ml) (Indoor Biotechnologies INC, Charlottesville, VA, USA) for 6 days at 5% CO_2_ and 37 °C conditions. In order to determine the Teff proliferative response, they were pre-stained with CFSE (5, 6-carboxifluoresceindiacetate N-succinimidyl ester) (Life Technologies, Eugene, OR, USA). During the last two hours of culture, GolgiSTOP (BD^TM^ Biosciences, San Jose, CA, USA) was added. Surface and intracellular characterization was carried out with specific MoAbs: CD3-PerCP, CD4-APC, CD4-APCH7 (BD™ Biosciences), CD27-PerCP and CD294-AF (Biolegend^®^). For intracellular cytokine staining, cells were fixed using the BD Cytofix/CytoPermFixation/Permeabilization solution kit (BD^TM^ Biosciences) and stained with IL-10-PE, IFN-γ-PECy7, IL-9-PE (BD^TM^ Bioscience) and IL-4-PECy7 (Biolegend^®^). T-cell subpopulations were defined as follows: CD3^+^CD4^+^IFN-γ^+^ (Th1); CD3^+^CD4^+^CD27^-^CD294^+^ (Th2); CD3^+^CD4^+^CD294^-^IL9^+^ (Th9). Production of IL-10, IL-4 and IL-9 cytokines was determined for all CD3^+^CD4^+^ cells. Cells were phenotyped using a BD^TM^ FACS Canto II flow cytometer (BD^TM^ Biosciences) and analyzed with FlowJo^®^ software (Tree Star). Dead cells were excluded using Near-IR fluorescent LIVE/DEAD^®^ Fixable Dead Cell dyes (Thermo Fisher Scientific).

Results are expressed as percentage of cells or as proliferation index (PI). This was calculated by dividing the percentage of CFSE^dim^ cells after Der p1 stimulation with the percentage of CFSE^dim^ cells without stimuli. A PI higher than 2 was considered positive^64–68^.

### Analysis of the cytokine mediated mechanism of Treg suppression

The effect on the proliferative response was determined using α-IL-10 (monoclonal mouse IgG_2B_) and α-TGF-β1 (monoclonal mouse IgG_1_) blocking antibodies (10 µg/ml) (R&D Systems, Minneapolis, MN, USA). These were added separately or together in all cell culture combinations described above and incubated for 6 days (5% CO_2_ and 37 °C). Surface and intracellular characterization was carried out as previously described and results expressed as PI.

### Statistical analysis

Data are presented as individual values and mean with s.d. or median and ranges as indicated. Qualitative variables were analyzed by chi-squared and Fisher tests. Quantitative variables were analyzed using the Mann-Whitney U-test. Comparisons of related samples were carried out by Wilcoxon test. Correlation analysis was performed by calculating the Pearson correlation coefficient. All reported p-values represented two-tailed tests, and p-values ≤ 0.05 are considered statistically significant.

The authors declare that all data supporting of this study are available within the paper and its supplementary files.

## Electronic supplementary material

Supplementary figure 1

Supplementary figure 2
