# Supplementary material for: Dermatophagoides pteronyssinus immunotherapy changes the T-regulatory cell activity
Source: Sci Rep. 2017 Sep 20;7:11949. doi: 10.1038/s41598-017-12261-2 (PMC5607227; doi:10.1038/s41598-017-12261-2)
Supplement: Supplementary file 1 — Supplementary figure 1 [file 41598_2017_12261_MOESM1_ESM.pdf]

TITTLE PAGE

Supplementary figure 1

Title: *Dermatophagoides pteronyssinus* immunotherapy changes the T-regulatory cell activity

Gonzalez M[1#], BsC; Doña I[2#], MD, PhD; Palomares F[1], PhD; Campo P[2], MD, PhD,; Rodriguez MJ[1], PhD; Rondon C[2], MD,PhD; Gomez F[2], MD, PhD; Fernandez TD[1], PhD; Perkins JR[1], PhD; Escribese MM[3, 4], PhD; Torres MJ[2*], MD, PhD; Mayorga C[1,2*], PhD.

[1]Research Laboratory-Allergy Unit, IBIMA-Regional University Hospital of Malaga, UMA, Malaga, Spain.

[2]Allergy Service, IBIMA-Regional University Hospital of Malaga, UMA, Malaga, Spain.

[3] Institute for Applied Molecular Medicine (IMMA), School of Medicine, Universidad CEU San Pablo, Madrid, Spain

[4]Basical Medical Sciences Department, Faculty of Medicine, CEU San Pablo University, Madrid, Spain

[#]Both authors contributed equally in this manuscript

* Both authors contributed equally in this manuscript

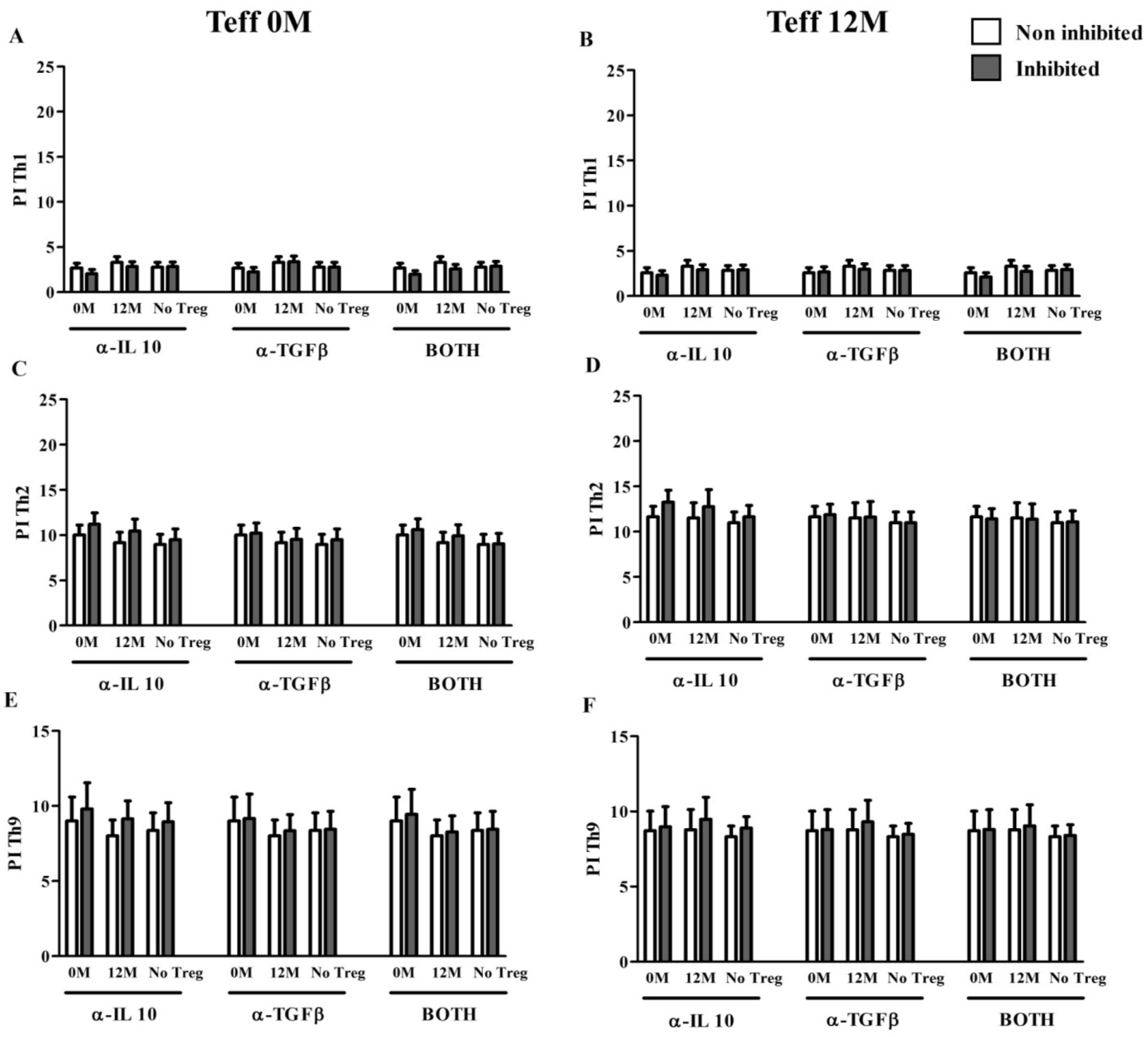

Teff 0M
Teff 12M
Non inhibited
Inhibited
A
B
C
D
E
F
PI Th1
PI Th2
PI Th9
0M
12M
No Treg
α-IL 10
α-TGFβ
BOTH
